# Experimental evidence for alleviating nociceptive hypersensitivity by single application of capsaicin

**DOI:** 10.1186/s12990-015-0019-0

**Published:** 2015-04-22

**Authors:** Xiao-Li Ma, Fang-Xiong Zhang, Fei Dong, Lan Bao, Xu Zhang

**Affiliations:** Institute of Neuroscience and State Key Laboratory of Neuroscience, CAS Center for Excellence in Brain Science, Shanghai Institutes for Biological Sciences, Chinese Academy of Sciences, 320 Yue Yang Road, Shanghai, 200031 China; State Key Laboratory of Cell Biology, Institute of Biochemistry and Cell Biology, Shanghai Institutes for Biological Sciences, Chinese Academy of Sciences, Shanghai, 200031 China; School of Life Science and Technology, ShanghaiTech University, Shanghai, 201210 China

## Abstract

The single application of high-concentration of capsaicin has been used as an analgesic therapy of persistent pain. However, its effectiveness and underlying mechanisms remain to be further evaluated with experimental approaches. The present study provided evidence showing that the single application of capsaicin dose-dependently alleviated nociceptive hypersensitivity, and reduced the action potential firing in small-diameter neurons of the dorsal root ganglia (DRG) in rats and mice. Pre-treatment with capsaicin reduced formalin-induced acute nocifensive behavior after a brief hyperalgesia in rats and mice. The inhibitory effects of capsaicin were calcium-dependent, and mediated by the capsaicin receptor (transient receptor potential vanilloid type-1). We further found that capsaicin exerted inhibitory effects on the persistent nociceptive hypersensitivity induced by peripheral inflammation and nerve injury. Thus, these results support the long-lasting and inhibitory effects of topical capsaicin on persistent pain, and the clinic use of capsaicin as a pain therapy.

## Background

Topical application of capsaicin has long been clinically used to treat persistent pain, such as osteoarthritic pain, post-herpetic neuralgia of the trigeminal nerve, migraine prophylaxis, diabetic neuropathy, HIV-associated distal sensory neuropathy, and intractable pain in cancer patients [1-9]. Capsaicin is an agonist of transient receptor potential cation channel, subfamily V, member 1 (TRPV1), which is expressed in small-diameter neurons of the dorsal root ganglion (DRG) [10]. Topical treatment with capsaicin in human initially results in nociceptor firing and a period of enhanced sensitivity to painful heat stimuli, and then a refractory period during which resistant to capsaicin and heat but not pinprick stimuli [4,11-14]. Application of capsaicin can induce long-lasting thermal hypoalgesia in the inflammatory model [15], and another TRPV1 agonist resiniferatoxin (RTX) alleviates the thermal nociception in the physiological state and under the inflammatory condition [16].

Neuronal TRPV1 is a homotetrameric, nonselective ligand-gated cation channel which can be activated by a wide range of stimuli, including heat, proton, exogenous compounds such as capsaicin and endovanilloids [17-19]. Studies with the TRPV1 gene knockout mice show that TRPV1 is involved in the nociceptive response induced by noxious heat (>50°C) and inflammation-induced thermal hyperalgesia, although responsiveness to noxious heat stimuli is not completely lost in TRPV1-deficient mice [20-22]. Capsaicin opens TRPV1 channel not only to increase the intracellular level of calcium and induce membrane depolarization, but also to initiate the desensitization and downregulation of TRPV1, and the degeneration of epidermal nerve fibers, which is referred as defunctionalization following continuous capsaicin exposure [5,23-26]. The long refractory period of the neurons which were excited previously by capsaicin may result from calcium-dependent conformational changes in TRPV1 protein, ultimately closing the channel pore [27]. However, this effect was presumably temporary and therefore might not account for a persistent pain relief observed clinically.

Studies on the mechanisms for long-lasting analgesia after continuous application of capsaicin suggest that the capsaicin treatment leads to the dysfunctionalization of TRPV1-containing nerve terminals in the skin and peripheral tissues, due to the long-term, functional and phenotypic alternation of neurons [26,28-31]. Capsaicin inhibits action potentials and voltage-gated sodium channels in capsaicin-sensitive DRG neurons, and repeated application of capsaicin produced membrane depolarization but failed to evoke action potentials [27,32-34]. Repeated treatment with capsaicin may reversibly decrease the density of epidermal nerve fibers, and thus the nociceptive deficiency could be induced several days after treatment and may last for weeks [4,5,24,26,29,35-38].

Recent clinical practice and study show that a single 60-min or 30-min application of 8% capsaicin patch was found to provide significant pain relief for at least 12 weeks in patients suffering persistent pain including peripheral neuropathic pain [30,31,39-43]. However, the experimental evidence for such an application of capsaicin remains to be obtained to evaluate its effectiveness and potential mechanisms for treatment of acute and chronic pain.

The present study showed that after a brief excitatory effect on capsaicin-sensitive DRG neurons and a transient nociceptive response of rats, a single application of capsaicin inhibited the action potential (AP) firing in capsaicin-sensitive DRG neurons and alleviated the nociceptive hypersensitivity in the acute pain model induced by formalin, the inflammatory pain model by complete Freund’s adjuvant (CFA) and neuropathic pain model by spared nerve injury (SNI). This inhibitory effect of capsaicin was mediated by TRPV1 channel, and appeared to be dependent on the dosage of capsaicin and calcium influx. These results provide a line of experimental evidence of the long-lasting and inhibitory effects of topical capsaicin on persistent pain, and support the clinic use of capsaicin as a pain therapy.

## Results

### Capsaicin pretreatment induces analgesic effect on the formalin-induced nociceptive response

**Figure 1:**
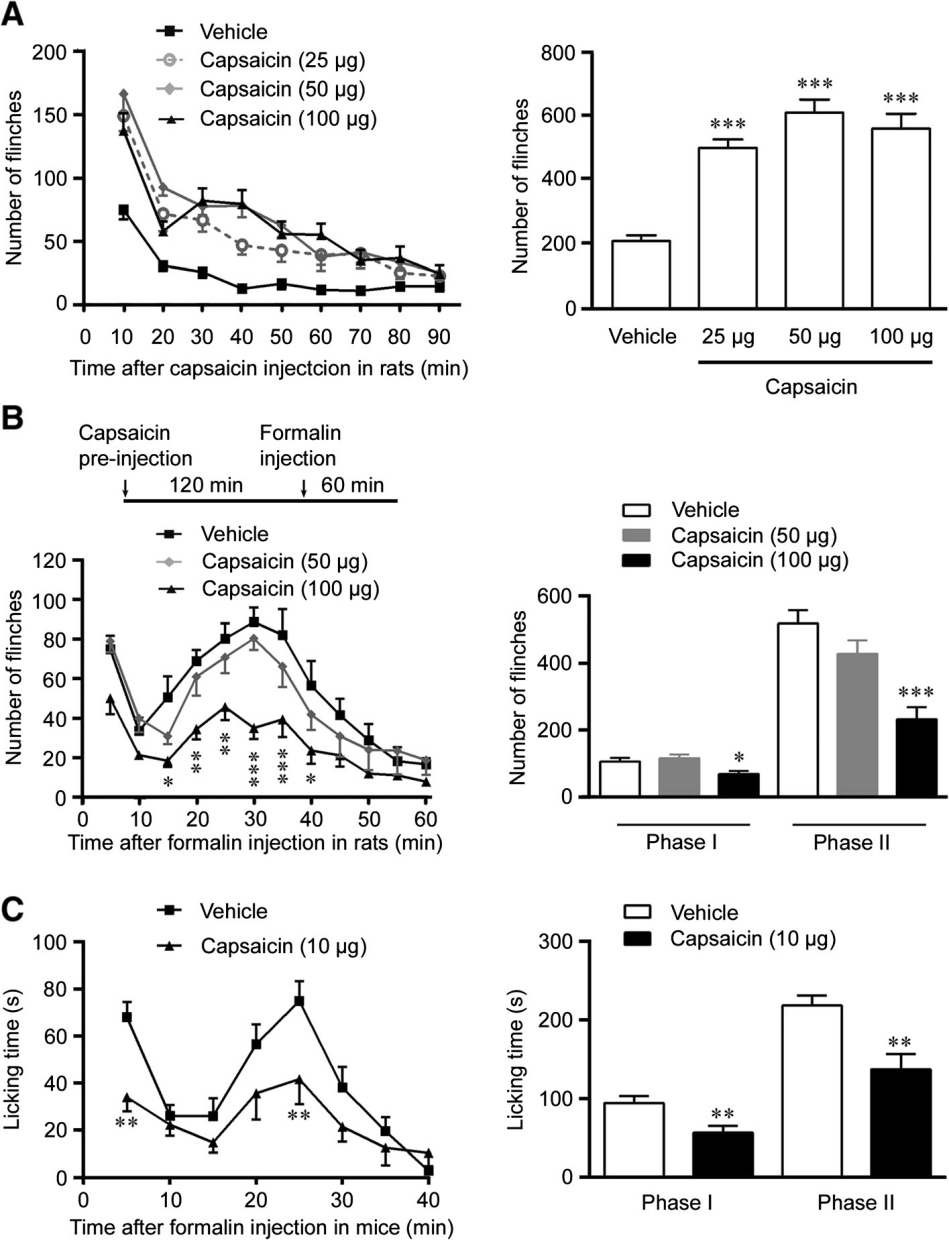
Capsaicin treatment causes acute algetic responses and a subsequent analgesic effect. **(A)** Time course of 90 min following intraplantar injection of 25 (n = 9 rats), 50 (n = 16) and 100 μg (n = 12) capsaicin or vehicle (n = 14) (p < 0.001 for 25 μg, p < 0.001 for 50 μg and p < 0.001 for 100 μg capsaicin versus vehicle control group, ANOVA). The quantitative analysis showed that the number of flinches was increased at capsaicin-injected hindpaw. **(B)** Intraplantar pre-injection of 100 μg capsaicin (n = 13) but not 50 μg capsaicin (n = 8) or vehicle (n = 12) induced an analgesic effect in the pain model induced by 2% formalin (p < 0.001 for 100 μg capsaicin versus vehicle group, ANOVA). The time course showed that the inhibitory effect of capsaicin on the formalin-induced flinch in the phase II was lasted for 30 min. The number of flinches in the phase I and phase II was reduced in the rats with capsaicin pre-treatment. **(C)** Intraplantar pre-injection of 10 μg capsaicin (n = 15) reduced the licking time of mice induced by 2% formalin, compared to vehicle group (n = 15) (p < 0.001, ANOVA). The licking time in both phase I and phase II was decreased in mice. Data are shown as mean ± SEM. *p < 0.05, **p < 0.01, ***p < 0.001, versus vehicle group.

We firstly examined the effect of capsaicin on the acute nociceptive response induced by intraplantar injection of formalin. As expected, the rats developed nocifensive behavior after receiving the capsaicin (25, 50 and 100 μg) injection at the hindpaw, as compared to the rats received the vehicle injection (Figure 1A). The flinch number of the hindpaw was increased to a peak level 10 min after the injection of 25, 50 and 100 μg capsaicin, and then gradually decreased 20–60 min after the capsaicin injection (Figure 1A). Most time points between vehicle group and capsaicin group showed significant difference (10, 20, 30, 40, 60 and 70 min between vehicle group and 25 μg group; 10, 20, 30, 40, 50 and 70 min between vehicle group and 50 μg group; 10, 30, 40, 50 and 60 min between vehicle group and 100 μg group). The increased flinch behavior disappeared 90 min after capsaicin injection.

Formalin injection caused a stereotypic two-phase pattern of nociceptive response shown by the increase in flinch number of the hindpaw. The phase I occurred within 0–10 min due to periphery nociceptive response, and the phase II appeared winthin 10–60 min due to central nociceptive reaction (Figure 1B). To examine the effect of capsaicin on acute pain, capsaicin (50 μg or 100 μg) was intraplantarly injected into the hindpaw 120 min before the formalin injection (Figure 1B). The formalin-induced nociceptive response in the phase I and phase II were both inhibited by the pretreatment with 100 μg capsaicin but not 50 μg capsaicin (Figure 1B), suggesting that the capsaicin pretreatment could dose-dependently prevent the formalin-induced acute nociceptive hypersensitivity. Similar to the rats, the formalin-induced nociceptive response in mice could be inhibited by the pretreatment with 10 μg capsaicin (Figure 1C).

### Capsaicin suppresses the excitability of small DRG neurons

**Figure 2:**
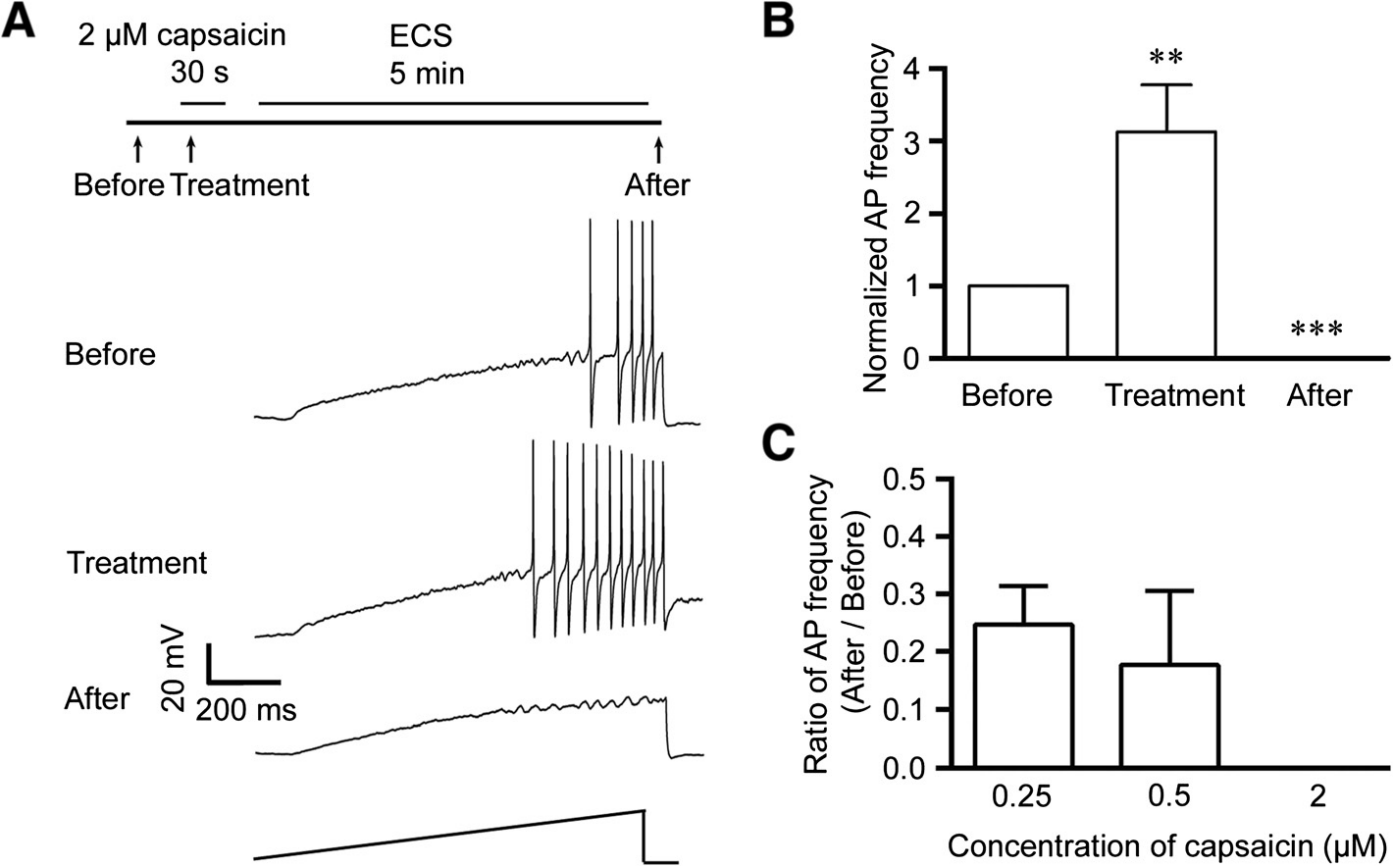
Pre-treatment with capsaicin reduces the excitability of small DRG neurons of rats. **(A)** Whole-cell patch-clamp recording in small neurons acutely dissociated from the rat DRGs showed that the firing frequency of action potentials induced by a current ramp (1 s duration; peak ranging from 100–500 pA) was firstly increased, and was then decreased after 5 min washout of capsaicin. **(B)** The action potential frequency during capsaicin treatment was increased to 3.12 (3.12 ± 0.65)-folds of that recorded before treatment, and such a change was attenuated in the same neuron after 5 min washout. **p < 0.01, ***p < 0.001, versus the same group of neurons before capsaicin treatment (n = 15 neurons). **(C)** The capsaicin-induced suppression of action potential was dose-dependent. The action potential frequency after 5 min washout reduced to 0.25-fold and 0.18-fold of that before 0.25 μM (n = 26) and 0.5 μM (n = 9) capsaicin treatment, respectively, and was almost totally suppressed with 2 μM capsaicin (n = 11).

To further examine whether the excitability of DRG neuron was regulated by capsaicin, we performed whole-cell patch-clamp recording in small neurons dissociated from the rat DRGs. The action potentials initiated by single current ramp stimulation were recorded before 30 s capsaicin treatment, during the treatment and 5 min washout after the treatment, respectively (Figure 2A). The TRPV1-positive neuron was recognized once an inward current was induced after puffing capsaicin. The action potential frequency in small DRG neurons was increased transitorily in response to the treatment with 2 μM capsaicin (Figure 2A and B), consistent with the acute nociceptive behavior following capsaicin treatment.

However, after washout with the extracellular solution (ECS) for 5 min, the same neurons no longer fired the action potentials in response to the same current ramp stimulation (Figure 2A and B). Furthermore, we found that this effect was dose-dependent, because 0.25 μM and 0.5 μM of capsaicin reduced the action potential frequency after 5 min washout to 0.25 (0.25 ± 0.07)-fold and 0.18 (0.18 ± 0.13)-fold of that before capsaicin treatment, respectively, while 2 μM of capsaicin completely suppressed the action potential firing (Figure 2C). At the same time interval, the membrane potential of the capsaicin-stimulated neurons returned to the basal levels, and the second treatment with 2 μM capsaicin could induce inward current with ~48% of the amplitude of first one (n = 5). Taken together, the treatment with capsaicin could result in a dose-dependent inhibitory effect on the excitability of small DRG neurons, consistent with the reduction in the nocicptive behavior after capsaicin pretreatment observed in rats.

### TRPV1 is required for the capsaicin-induced inhibition

**Figure 3:**
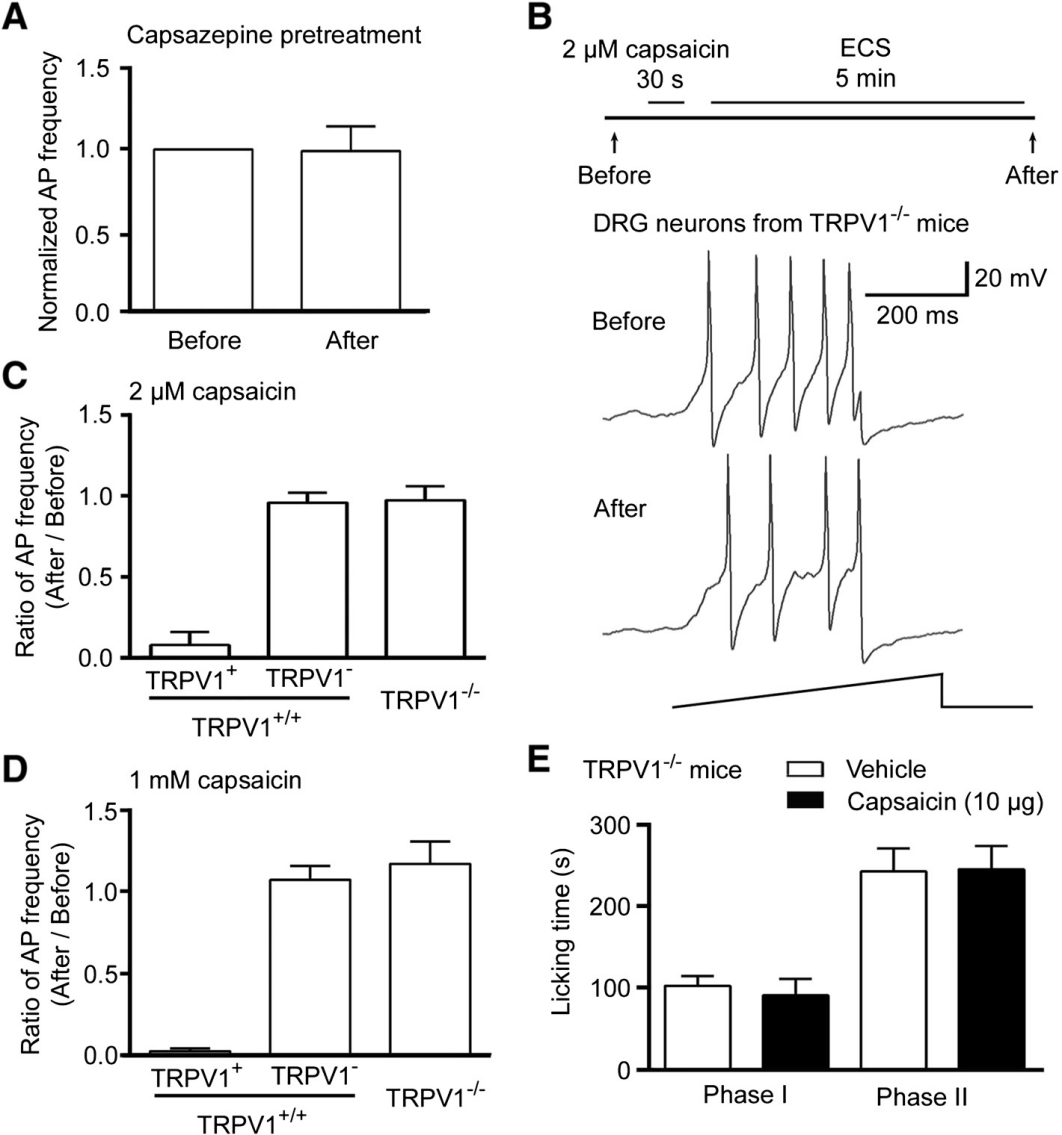
TRPV1 is required for the inhibitory effect of capsaicin on the neuronal excitability and formalin-induced nociceptive response. **(A)** Treatment with TRPV1 antagonist, capsazepine, attenuated the capsaicin-induced reduction in action potential frequency in small DRG neurons (n = 13). **(B**, **C**, **D**) Whole-cell patch-clamp recording showed that the capsaicin-induced reduction of action potential frequency in capsaicin-sensitive small DRG neurons (for 2 μM capsaicin, 10 positive neurons out of 34; for 1 mM capsaicin, 8 positive neurons out of 22) dissociated from TRPV1^+/+^ littermates was absent in small DRG neurons from TRPV1^−/−^ mice (for 2 μM capsaicin, n = 31; for 1 mM capsaicin, n = 10). **(E)** The licking time in both phase I and phase II of mice induced by 2% formalin was not affected by intraplantar pre-injection of 10 μg capsaicin (n = 8), compared to vehicle group (n = 8). Data are shown as mean ± SEM.

To examine whether this capsaicin-induced inhibition was specifically mediated via TRPV1, neurons dissociated from the rat DRGs were incubated with capsazepine, the antagonist of TRPV1, for 30 min before the treatment with 2 μM capsaicin. This pre-blockade of TRPV1 prevented the capsaicin-induced reduction in the action potential frequency after 5 min washout in small DRG neurons (Figure 3A). Furthermore, we performed whole-cell patch-clamp recording in small DRG neurons dissociated from the DRGs of TRPV1 gene knock-out (TRPV1^−/−^) and wild-type littermate (TRPV1^+/+^) mice. The 30 s pretreatment with 2 μM capsaicin and following 5 min washout in the capsaicin-sensitive small neurons (n = 10/34) from TRPV1^+/+^ mice reduced the action potential frequency to 0.08 (0.08 ± 0.08)-fold of that before capsaicin treatment, while the capsaicin-insensitive neurons had no obvious changes (Figure 3B and C). In contrast, the small DRG neurons (n = 31) from TRPV1^−/−^ mice did not show any changes in the action potential frequency (Figure 3B and C). We also examined the effect of capsaicin at a higher concentration. The 30 s pretreatment with 1 mM capsaicin and following 5 min washout reduced the action potential frequency to 0.02 (0.02 ± 0.02)-fold of that before capsaicin treatment in the capsaicin-sensitive small neurons (n = 8/22) from TRPV1^+/+^ mice, while the capsaicin-insensitive neurons from wild-type mice and the small DRG neurons (n = 10) from TRPV1^−/−^ mice did not show any changes in the action potential frequency (Figure 3D). Thus, TRPV1 in small DRG neurons is required for the capsaicin-induced inhibition of excitability.

We further examined the effect of capsaicin on the formalin-induced nociceptive response in TRPV1^−/−^ mice. The capsaicin-induced inhibition on the phase I and phase II of the formalin-induced nociceptive response was lost in TRPV1^−/−^ mice (Figure 3E). These data suggest that TRPV1 is required for the capsaicin-induced inhibition.

### Capsaicin-induced inhibition is calcium-dependent

**Figure 4:**
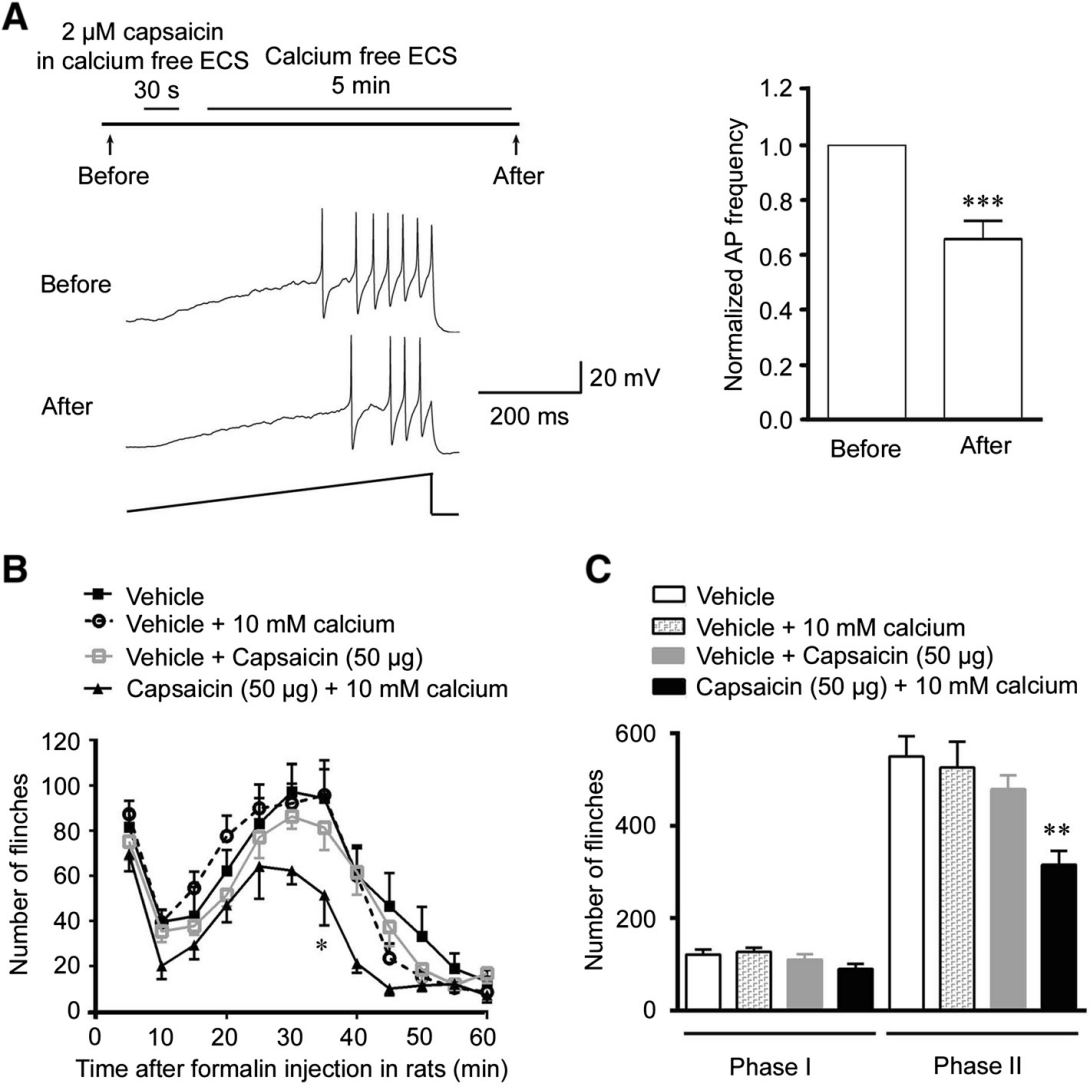
Calcium is involved in the capsaicin-induced inhibition on neuronal excitability and analgesia. **(A)** whole-cell patch-clamp recording showed that in the absence of extracellular calcium, the ratio of action potential frequency was only partially inhibited in capsaicin-pretreated small DRG neurons. ***p < 0.001, versus the same group of neurons before capsaicin treatment (n = 30). **(B**, **C)** Behavior test showed that 50 μg capsaicin or 10 mM calcium or vehicle (n = 9) alone could not reduce the nociceptive response in the phase II of formalin test. However, the injection of a mixture of 50 μg capsaicin with 10 mM calcium (n = 7) could inhibit the reaction in the phase II (p < 0.001 for rats treated with capsaicin mixed with 10 mM calcium versus vehicle group, ANOVA). Data are shown as mean ± SEM. **p < 0.01, versus vehicle group.

Considering TRPV1 as a non-selective cation channel, we next asked whether calcium influx induced by activation of TRPV1 was involved in the capsaicin-induced analgesia. Small DRG neurons dissociated from rats were incubated in the calcium-free ECS, and were then stimulated by a current ramp. We found that in the absence of extracellular calcium, 2 μM capsaicin treatment reduced the action potential frequency after 5 min washout to 0.66 (0.66 ± 0.07)-fold of that before capsaicin treatment (Figure 4A). This result suggests that the influx of extracellular calcium is required for the capsaicin-induced inhibition on nociceptive afferent neurons.

To test whether the extracellular calcium affect the capsaicin-induced analgesia, 50 μg capsaicin was mixed with 10 mM calcium was injected 120 min before formalin injection. This mixture significantly reduced the nociceptive response in the phase II of formalin test and also caused a tendency of decreased response in the phase I, whereas 50 μg capsaicin and 10 mM calcium alone did not affect the nociceptive response (Figure 4B and C). Taken together, the capsaicin-induced inhibition on the nociceptive response could be regulated by the influx of extracellular calcium.

### Capsaicin reduces the nociceptive hypersensitivity induced by peripheral inflammation and nerve injury

**Figure 5:**
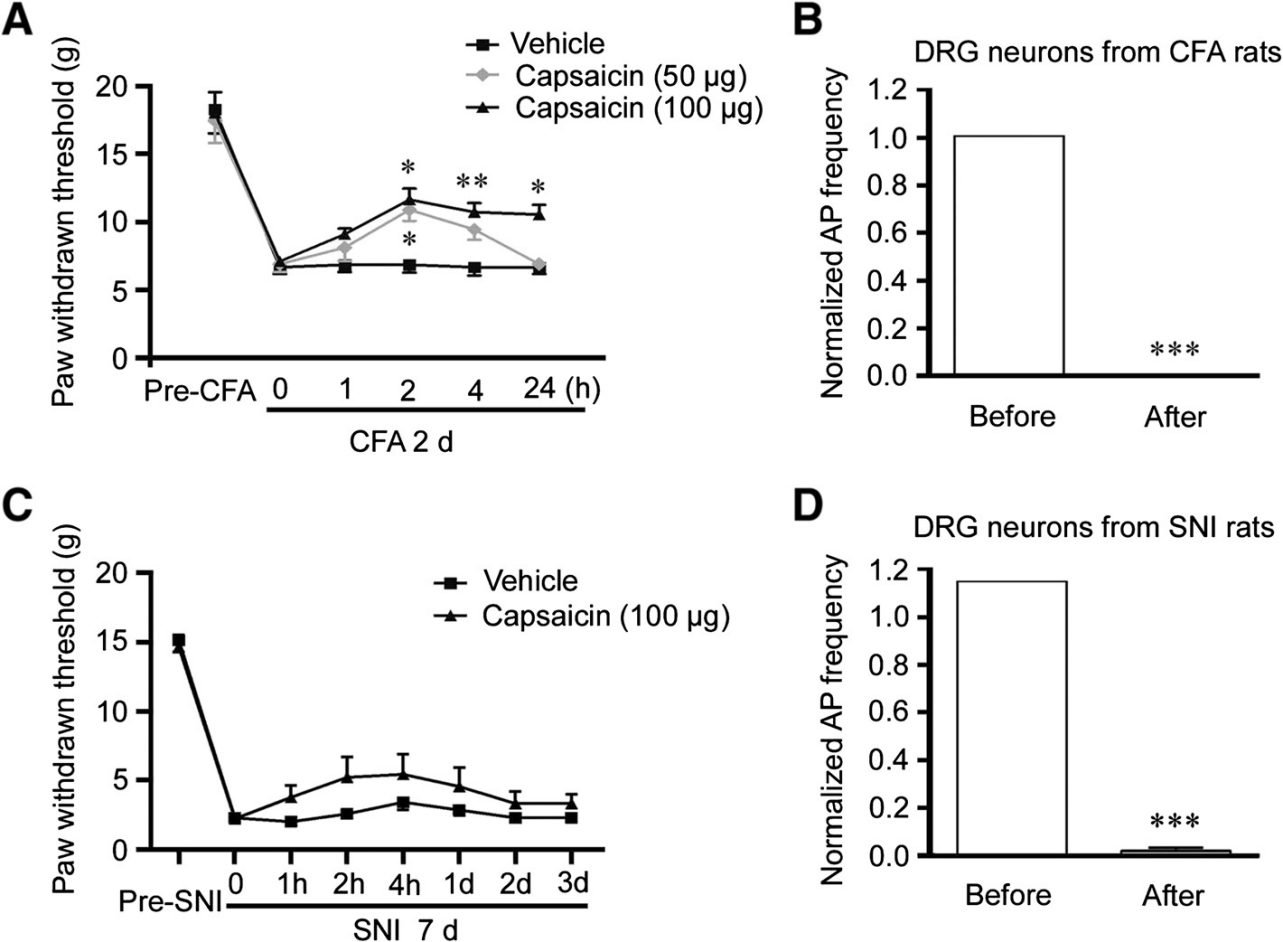
Capsaicin alleviates the nociceptive hypersensitivity induced by peripheral inflammation or nerve injury. **(A)** Intraplantar injection of 100 μg capsaicin could reduce the mechanical allodynia 2 days after CFA injection (n = 12 for vehicle, n = 9 for 50 μg capsaicin treatment and n = 11 for 100 μg capsaicin treatment). The significant analgesic effect of 100 μg capsaicin began 2 h after capsaicin injection and maintained for 24 h, while 50 μg showed a shorter analgesic effect (p < 0.05 for 50 μg and p < 0.001 for 100 μg capsaicin versus vehicle group, ANOVA). **(B)** Whole-cell patch-clamp recording showed that puffing 2 μM capsaicin for 30 s, the action potential frequency was totally suppressed after 5 min washout in small DRG neurons dissociated from rats injected CFA for 2 days. ***p < 0.001, versus the same group of neurons before capsaicin treatment (n = 11). **(C)** Intraplantar injection of 100 μg capsaicin could mildly reduce mechanical allodynia 7 days after SNI (n = 8 for vehicle and n = 9 for capsaicin treatment) (p < 0.01 for 100 μg capsaicin versus vehicle group, ANOVA). **(D)** Whole-cell patch-clamp recording showed that treatment with 2 μM capsaicin for 30 s almost totally suppressed the action potential firing after 5 min washout in small DRG neurons dissociated from rats on post-SNI day 7. ***p < 0.001, versus the same group of neurons before capsaicin treatment (n = 17). Data are shown as mean ± SEM.

Using chronic inflammatory pain model induced by CFA and the neuropathic pain model induced by SNI, we evaluated whether capsaicin could alleviate persistent mechanical allodynia. The von Frey test was used to measure the mechanical threshold of rats. The mechanical allodynia developed 2 days after intraplantar injection of CFA at left hindpaw. We found that the intraplantar injection of 50 μg capsaicin reduced mechanical allodynia in subsequent 2 h compared to the vehicle-injected control group, while the analgesic effect of 100 μg capsaicin lasted for at least 24 h (Figure 5A). Furthermore, small DRG neurons dissociated from the CFA-treated rats were recorded to examine the neuronal excitability. A ramp current was injected into the neuron to induce action potential before and after the capsaicin application. After puffing 2 μM capsaicin for 30 s, the action potential frequency was completely suppressed after 5 min washout (Figure 5B).

We also evaluated the effect of capsaicin treatment on the mechanical allodynia in the SNI model. Seven days after SNI, the intraplantar injection of 100 μg capsaicin could mildly reduce the mechanical allodynia (Figure 5C). Whole-cell patch-clamp recording showed that in small DRG neurons dissociated from rats on post-SNI day 7, 2 μM capsaicin for 30 s almost completely suppressed the action potential frequency after 5 min washout (Figure 5D). Taken together, capsaicin treatment alleviates the persistent nociceptive hypersensitivity induced by peripheral inflammation and nerve injury.

## Discussion

The present study showed that a brief and topical capsaicin treatment could lead to long-lasting inhibition of neuronal excitability in a dose- and calcium-dependent manner, and alleviation of both acute and persistent nociceptive responses. These results suggest that a single application of capsaicin could produce a therapeutic approach to effectively treat persistent pain.

Clinical practice suggests that capsaicin can be used as an analgesic to relief pain [1-9]. Particularly, recent reports show that a single 60-min or 30-min application of 8% capsaicin patch significantly relieves pain for many weeks in patients [30,31,39-43]. However, the experimental evidence for the capsaicin-induced analgesia and the related mechanism has been insufficient to support this clinical application of capsaicin. In the present study, we analyzed the analgesic effect of capsaicin in the animal models of acute and chronic pain, including the formalin test, CFA model of chronic inflammatory pain and SNI model of chronic neuropathic pain. Our results showed that a single application of capsaicin reduced the nociceptive hypersensitivity induced by acute noxious stimulation and peripheral inflammation or nerve injury. Notably, the analgesic effect of capsaicin was more pronounced in the inflammation model than that in the neuropathic pain model, which may be due to the elevated expression of TRPV1 in inflammatory condition and the reduced expression of TRPV1 in neuropathic pain model [44,45]. Thus, the single injection of capsaicin may be more effective in managing inflammatory pain than neuropathic pain.

Previous studies showed that capsaicin desensitizes capsaicin-sensitive nociceptors by inhibiting the generation of action potentials through the indirect block of voltage-gated sodium channels [27,32-34]. In the present study, we found that a brief treatment with capsaicin could induce long-lasting suppressive effects on the generation of action potentials in capsaicin-sensitive DRG neurons in a dose- and calcium-dependent manner, consistent with the inhibitory effects of a single capsaicin treatment on the acute and persistent nociceptive hypersensitivity. Our finding that high-dose capsaicin produced more pronounced inhibition is consistent with the clinical observation that high-concentration topical capsaicin is required to treat neuropathic pain [30,31,39,40,43,46]. Our present results show that the capsaicin-induced calcium influx involves the acute regulation of both neuronal excitability and nociceptive behavior induced by a single injection of capsaicin. This is consistent with the notion that the long refractory period of the neurons after capsaicin treatment may result from calcium-dependent conformational changes in TRPV1 protein [27], suggesting that the refractory period of capsaicin-sensitive neurons also accounts for the relief of persistent pain.

The refractory period of capsaicin-sensitive neurons could be attributed to a desensitization state of TRPV1 or the depolarized state of DRG neuron after TRPV1 opening [2,42]. The present electrophysiological experiments showed that there were a short period of TRPV1 desensitization and membrane depolarization following capsaicin treatment. However, after 5 min washout, DRG neurons were re-sensitized to capsaicin and the membrane potential returned to the basal levels, whereas the neuronal excitability at the same time interval was suppressed. Therefore, the analgesia induced by a single application of capsaicin might be not attributed to the TRPV1 desensitization or the membrane depolarization of sensory neurons. An alternative explanation could be a reduced excitability of primary sensory neurons. Capsaicin was found to inhibit the generation of action potentials and voltage-gated sodium channels in the DRG neurons that were previously excited by capsaicin [27,32-34]. Moreover, capsaicin induces sodium influx, which would indirectly induce the membrane depolarization and inactivation of voltage-gated sodium channels [32-34]. Therefore, a single application of capsaicin may desensitize nociceptors by inhibiting the generation of action potentials.

Previous studies showed that heat responses, but not mechanical hyperalgesia or allodynia, were attenuated by ablation of TRPV1-lineage neurons using genetic approach in mice [47]. TRPV1 may distribute in both peptidergic and non-peptidergic neurons of rats [48]. Selective blocking sodium channels in the TRPV1-expressing neurons of rats showed reduced response to both noxious mechanical and thermal stimuli [49]. We found that intraplantar injection of capsaicin attenuated mechanical hyperalgesia in the CFA model and mechanical allodynia in SNI model of the rat. One explanation is that high-dose capsaicin activated TRPV1 and then indirectly suppressed the sodium channels in nociceptive afferent neurons to reduce mechanical hypersensitivity.

Taken together, the present study showed that the generation of action potentials was attenuated in the capsaicin-sensitive DRG neurons that were previously excited by a single and brief treatment of capsaicin. A single injection of capsaicin significantly reduced the nociceptive hypersensitivity in both the rat model of peripheral inflammation and the model of neuropathic pain. However, its effect on the inflammatory nociceptive response is more pronounced than that in the neuropathic pain model. Thus, our study provides experimental evidence supporting the single application of high-dose capsaicin as a clinical approach to treat persistent pain.

## Methods

### Animals and drugs

All experiments were approved by the Committee of Use of Laboratory Animals and Common Facility, Institute of Neuroscience, Chinese Academy of Sciences and carried out according to the guidelines of the International Association for the Study of Pain. All animals were housed under a 12-h light/dark cycle at 22-26°C, with free access to food and water. Sprague–Dawley male rats were bought from Shanghai SLAC Laboratory Animal CO. LTD (SLAC). TRPV1 knockout (TRPV1^−/−^) mice were bought from the Jackson Laboratory (JAX). The strain name is B6.129X1-TRPV1^tm1jul^/J and the stock number is 003770. An exon encoding part of the fifth and all of the sixth putative trans-membrane domains together with the pore-loop region was disrupted [20]. The heterozygotes were bred to obtain TRPV1^−/−^ mice and their wild-type littermates (TRPV1^+/+^). TRPV1^−/−^ mice were viable and fertile. Capsaicin and capsazepine were purchased from Tocris Bioscience.

### Animal models and behavior tests

For acute nociceptive response of capsaicin, rats were divided into several groups after habituated in Perspex chamber with a wire mesh floor for at least 2 h, observed in the chamber, and then injected with vehicle or different dose of capsaicin intraplantarly. The number of flinches was counted every 10 min during 90 min after injection.

For tonic inflammatory formalin test, rats were habituated in Perspex chamber for at least 2 h. The rats were intraplantarly pretreated with vehicle or capsaicin for 2 h. After that, 50 μl of 2% formalin (Sigma-Aldrich) in saline was injected into the rat left hindpaw intraplantarly. The number of flinches was counted based on the first phase (1–10 min) and the second phase (10–60 min) [50,51].

For formalin test in TRPV1^+/+^ and TRPV1^−/−^ mice, the mice were intraplantarly pretreated with vehicle or 10 μg capsaicin for 2 h. Then, mice were injected with 20 μl of 2% formalin into the dorsal surface of the left hindpaw. The time that mice licked the injected paw was recorded during the phase I (1–10 min) and II (10–40 min).

As chronic inflammatory pain model, rats were injected with 100 μl CFA (Sigma-Aldrich) at the hindpaw intraplantarly. To determine the threshold for mechanical pain response, the von Frey test was performed 2 days after injection. For electrophysiological experiment, CFA-treated rats were sacrificed 2 days after injection.

As chronic neuropathic pain model, the SNI model was performed on Sprague–Dawley rats as previously described. Two of the three branches of the sciatic nerve (common peroneal nerve and tibial nerve) were injured, leaving the remaining sural nerve intact [52,53]. The von Frey test and electrophysiological experiment were performed on 7 d after SNI surgery.

To measure mechanical threshold, the rats were habituated in Perspex chamber for more than 2 h before tests, and then graded von Frey filaments were applied to the lateral area of the hindpaw, using the up-and-down testing paradigm [54]. The minimum bending force with three positive responses out of five stimuli with von Frey filaments presented perpendicular to the plantar surface of the rat hindpaw was determined as the mechanical threshold.

All behavioral tests were all performed double-blindly.

### Cell culture and electrophysiology

L4 and L5 DRGs were acutely dissected from approximately 100 g Sprague–Dawley rats or approximately 20 g TRPV1^+/+^ and TRPV1^−/−^ mice after they were anesthetized by injection of pentobarbital sodium (80 mg/kg). The connective tissue was digested by 0.4 mg/ml trypsin type I (Sigma-Aldrich), 1 mg/ml collagenase type 1A (Sigma-Aldrich), and 0.1 mg/ml DNase I (Sigma-Aldrich) in DMEM Medium (Gibco-Invitrogen, Carlsbad, CA, USA) at 37°C followed by three washes in ECS. DRGs were then mechanically dissociated with a set of flame-polished Pasteur pipettes. Dissociated cells were placed on glass coverslips at room temperature and patch clamp recording was performed within 2–12 h.

Neuronal excitability was examined with long suprathreshold current pulse (1 s). The amount of injecting current (100–500 pA) was individually determined for each neuron by repeated injections that evoke multiple action potentials. The TRPV1-positive neurons were selected by observing immediate inward current when capsaicin was applied. Once an inward current was induced after puffing capsaicin, we recognized this neuron as a TRPV1-positive one. To compare the effect of single application of capsaicin on neuronal excitability, the number of action potential was normalized to that before capsaicin treatment for each neuron. The ratio of action potential frequency was calculated from the number of action potential after 5 min washout versus that before capsaicin treatment for each neuron.

Normal ECS contained following: 150 mM NaCl, 5 mM KCl, 2.5 mM CaCl_2_, 1 mM MgCl_2_, 10 mM HEPES and 10 mM glucose, then adjusted to pH 7.4 with NaOH. To prepare ECS containing 0 mM Ca^2+^, 2.5 mM CaCl_2_ was replaced by 2.5 mM MgCl_2_. Electrodes had a resistance of 2–4 MΩ when filled with the pipette solution, which contained the following: 140 mM KCl, 1 mM MgCl_2_, 2.5 mM CaCl_2_, 5 mM EGTA, 10 mM HEPES, 2 mM Na-ATP and 0.3 mM Na-GTP, then adjusted to pH 7.3 [55]. Whole-cell recordings were performed if recorded neurons matched following criteria: small-diameter DRG neuron with < 30 pF capacitance, 1 GΩ seal resistance before “breakthrough” with additional suction and 400 MΩ input resistance in whole-cell mode. Once whole-cell access was obtained, the current clamp mode was switched after 5 min balance. Data were collected using an EPC-9 patch-clamp amplifier and the Pulse software (version 8.31; HEKA Elektronik). Action potential parameters were analyzed with MATLAB program.

### Statistical analysis

Statistic analyses were performed using Graphpad Prism software (Graphpad software). Comparison of values between two groups was performed by Student’s pair t test or unpaired t test. Difference for behavior data were compared with two way ANOVA followed by *post hoc* test. Difference was considered to reach statistical significance when p < 0.05. Levels of significance are indicated by the number of p value, e.g., *, 0.01 < p < 0.05; **, 0.001 < p < 0.01; ***, p < 0.001. Data are present as mean ± SEM.

## Abbreviations

AP: Action potential
CFA: Complete Freund’s adjuvant
SNI: Spared nerve injury
DRG: Dorsal root ganglion
ECS: Extracellular solution
RTX: Resiniferatoxin
TRPV1: Transient receptor potential cation channel, subfamily V, member 1
TRPV1^−/−^: TRPV1 gene knock-out
TRPV1^+/+^: TRPV1 wild-type
